# Can Progressive Strength Training Counteract Frailty and Improve Short-Term Autonomic Compensatory Responses During Active Standing Orthostatic Stress? A Pilot Study

**DOI:** 10.3390/jcm15051679

**Published:** 2026-02-24

**Authors:** Dihogo Gama de Matos, Jefferson Lima de Santana, Felipe J. Aidar, Stephen M. Cornish, Gordon G. Giesbrecht, Albená Nunes-Silva, Roman Romero-Ortuno, Todd A. Duhamel, Rodrigo Villar

**Affiliations:** 1 Cardiorespiratory & Physiology of Exercise Laboratory, Faculty of Kinesiology and Recreation Management, University of Manitoba, Winnipeg, MB R3T 2N2, Canada; 2 Graduate Program in Physical Education and Kinesiology and Physiological Sciences, Federal University of Sergipe, Sao Cristovao 49100-000, Brazil; 3 Centre on Aging, University of Manitoba, Fort Garry Campus, Winnipeg, MB R3T 2N2, Canada; stephen.cornish@umanitoba.ca; 4 Faculty of Kinesiology and Recreation Management, University of Manitoba, Winnipeg, MB R3T 2N2, Canada; 5 Departments of Anesthesia and Emergency Medicine, University of Manitoba, Winnipeg, MB R3T 2N2, Canada; 6 Laboratory of Inflammation and Exercise Immunology, Department of Physical Education, School of Physical Education, Federal University of Ouro Preto, Ouro Preto 35400-000, Brazil; 7 Discipline of Medical Gerontology, School of Medicine, Trinity College Dublin, D02 PN40 Dublin, Ireland; 8 Institute of Cardiovascular Sciences, St. Boniface General Hospital Albrechtsen Research Centre, Winnipeg, MB R2H 2A6, Canada; 9 Department of Physiology and Pathophysiology, University of Manitoba, Winnipeg, MB R3E 0J9, Canada

**Keywords:** autonomic function, frailty, older adults, heart rate variability, cardiac parasympathetic modulation, baroreceptor gain, strength training

## Abstract

**Background**: Frailty is a multifactorial condition that significantly impacts older adults’ health and independence, which can be mitigated through training. This study examined the effects of a 12-week progressive strength training (PST) program on frailty status and short-term autonomic compensatory responses during postural transitions. **Methods**: Eight older adults (60–79 years) classified as pre-frail or frail according to the frailty index (FI) participated in a 12-week PST program. Time and frequency-domain heart rate variability (HRV) in the supine position, cardiac parasympathetic modulation (CPM) determined from the HR 30:15 ratio (longest RR interval around the 30th heartbeat divided by the shortest RR interval around the 15th heartbeat after standing), and cardiac baroreceptor gain (CBG) assessed as the ratio of heart rate change to systolic blood pressure drop (ΔHR/ΔSBP) at 30, 60, 180, and 420 s after standing were assessed at pre-test, 8 weeks and 12 weeks (autonomic function outcomes). Physical activity levels (PAL), handgrip strength (HGS), and gait speed (GS) were assessed, and orthostatic intolerance (OI) symptoms were self-reported at pre-test, 8 weeks, and 12 weeks. **Results**: After 12 weeks of PST, FI scores decreased from 0.18 to 0.04 (78% reduction). PAL, HGS, and GS improved by 152%, 13%, and 11%, respectively. Three of eight participants reported OI symptoms at pre-test, with no reported symptoms at week 12. Despite this, PST did not enhance short-term autonomic responses. **Conclusions**: PST counteracted frailty and improved physical and muscular function but did not enhance indices of short-term autonomic regulation in frail older people.

## 1. Introduction

Frailty is a dynamic and multifactorial condition that significantly impacts the health and independence of older adults [1]. Although it can be mitigated through targeted interventions such as progressive strength training (PST) [2,3], the majority of studies have primarily demonstrated improvements using frailty identification instruments such as the frailty phenotype or the frailty index [4,5,6]. Whilst these instruments may capture symptoms, morbidities, disabilities and/or aspects of physical and functional status, they do not directly measure the underlying physiological mechanisms. As a result, while favourable exercise-induced transitions in frailty status have been well-documented [4], it remains unclear whether these positive changes stem from enhanced autonomic nervous system regulation, dysfunction of which is frequently associated with frailty.

The autonomic nervous system (ANS) is essential for regulating autonomic responses and ensuring rapid physiological adjustments during daily stressors such as postural transitions (e.g., from lying to standing; sitting to standing) [7]. If the ANS is not well-regulated, people could have orthostatic hypotension (OH), a condition characterized by a sudden drop in blood pressure upon standing [8]. OH is a key contributor to orthostatic intolerance (OI) [9] (e.g., lightheadedness, faintness, blurry vision), increasing instability in the first few seconds on standing, and the risk of falls [10], which has also been linked with frailty [11].

One frequently used non-invasive measurement of autonomic function is heart rate variability (HRV) [12], which reflects the beat-to-beat fluctuations of the time intervals between successive heartbeats [13]. However, the literature remains inconsistent regarding the effects of PST on HRV. For example, Lin et al. [14] found significant changes in HRV after 24 weeks of strength training in older adults (females and males), showing that individual differences in baseline autonomic function, age-related physiological alterations, and health status may influence the autonomic adaptations after strength training. In contrast, Gambassi et al. [15] reported no significant improvements in HRV following a 10-week strength training program in older females, suggesting impaired parasympathetic modulation. Cardiac parasympathetic modulation (CPM) is another important factor that provides an index of cardiac parasympathetic withdrawal on standing [16]. There is a lack of literature regarding the specific effects of PST on CPM in frail older adults, limiting our understanding of how exercise might improve autonomic regulation in this population. Another marker of autonomic function is cardiac baroreceptor gain (CBG), which reflects the sensitivity of the baroreflex in modulating heart rate (HR) in response to blood pressure (BP) fluctuations [17]. Despite its relevance, only a few studies have addressed how strength training influences CBG [18,19]. However, no study has examined the effects of PST on CBG across multiple specific short-term compensatory responses following postural transitions (e.g., 30 s, 60 s, 180 s, and 420 s) in frail individuals. Assessing the PST effects on CBG in this time-course framework could show novel insights into the temporal dynamics of autonomic adaptation to PST in frail older adults.

This pilot study aimed to (1) determine whether a 12-week PST program can counteract frailty; (2) examine changes in short-term autonomic responses, including HRV, CPM, and CBG, across postural transitions; and (3) evaluate changes in physical activity levels (PAL), handgrip strength (HGS), and gait speed (GS), as well as OI symptoms following 12 weeks of PST. It is hypothesized that PST (*i*) reduces frailty index scores, indicating improved frailty status; (*ii*) enhances resting HRV in the supine position, reflecting improved autonomic adaptability; (*iii*) increases CPM, indicating lower parasympathetic dominance and greater autonomic responsiveness; (*iv*) increases CBG across postural phases (30, 60, 180, and 420 s), suggesting faster baroreceptor activation; (*v*) improves PAL, HGS, and GS; and (*vi*) reduces the incidence of OI symptoms after 12 weeks of PST.

## 2. Materials and Methods

This study employed a one-group time series quasi-experimental design. Reporting followed established guidelines, including the Transparent Reporting of Evaluations with Non-Randomized Designs (TREND) [20], the Consensus on Exercise Reporting Template (CERT) [21], and the Consolidated Standards of Reporting Trials (CONSORT)—Extension for Pilot and Feasibility Trials [22].

**Settings**

The study took place at the Applied Research Centre within the Faculty of Kinesiology and Recreation Management at the University of Manitoba from August 2022 to August 2023. Ethical clearance was granted by the University of Manitoba Health Research Ethics Board (Protocol No. HE2022-0058).

**Participants**

**Figure 1:**
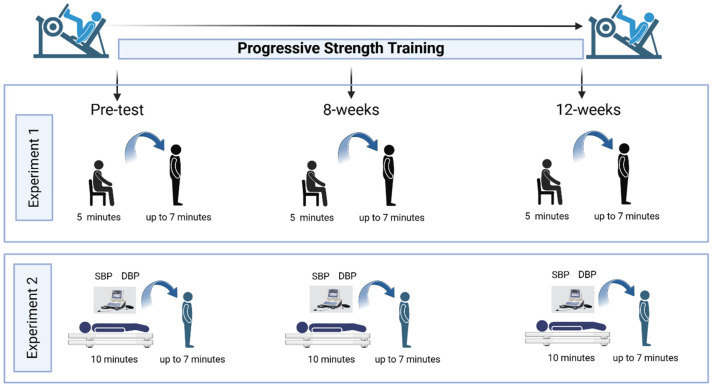
Progressive strength training program timeline.

A convenience sampling approach was utilized to recruit pre-frail and frail older adults. Potential participants were identified from Dr. Todd Duhamel’s database registry at the University of Manitoba, including over 1000 female volunteers from the Women’s Advanced Risk-Assessment in Manitoba (WARM) Hearts study and our Living F.R.E.E (Frailty Research in ExercisE) database; all had previously consented to be contacted by email or telephone. Individuals interested in participating could also reach out to the research team via email or telephone. The final sample included adults aged 60 to 79 years who completed a 12-week PST intervention and underwent orthostatic stress assessments involving sit-to-stand and lie-to-stand transitions (Figure 1).

Inclusion criteria required participants to be within the target age range (60–79 years), assigned female or male at birth, and classified as pre-frail or frail according to the 30-item frailty. The FI was created according to the standard procedure based on the deficit accumulation model [23]. Exclusion criteria included any history of cardiovascular disease (e.g., ischemic heart disease, myocardial infarction, stroke, percutaneous coronary intervention, coronary artery bypass grafting, or congestive heart failure), psychiatric disorders, or significant cognitive impairment. None of the participants was receiving hormone replacement therapy.

**Figure 2:**
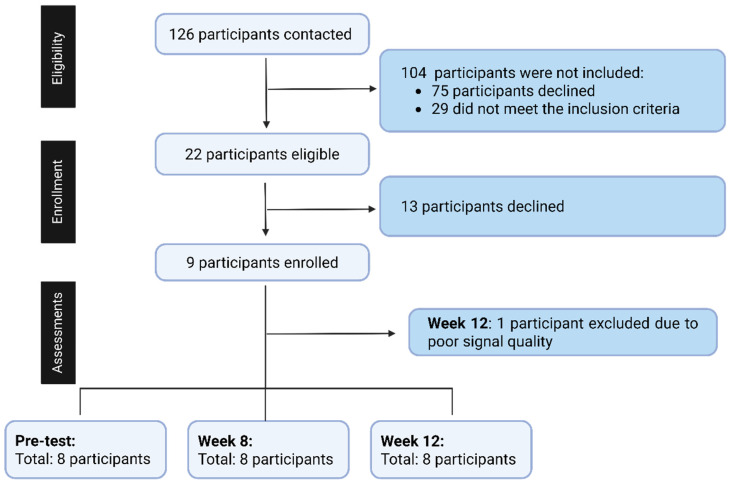
Flow chart of the participants’ recruitment.

Participant eligibility was confirmed through a health screening questionnaire developed by the research team to ensure adherence to all inclusion and exclusion criteria. The recruitment process is summarized in Figure 2. Twenty-two pre-frail or frail individuals were contacted; 13 participants declined participation, and nine participants were initially enrolled and completed assessments at pre-test, 8 weeks, and 12 weeks. One participant was excluded from the final 12-week analysis due to poor signal quality resulting from excessive noise and motion artifacts. To maintain the assumptions required for within-subject repeated-measures analysis and avoid missing data, the final analysis included eight participants with complete data across all time points.

**Data Collection**

Participants were provided with pre-assessment instructions 48 h before their scheduled visit to the laboratory. Upon arrival, they were given the informed consent form to review, and any questions were addressed by the research team before obtaining written consent. Before initiating any testing procedures, a trained researcher measured resting HR and BP to confirm that participants met the safety criteria required for participation. Resting systolic and diastolic blood pressure (SBP and DBP) readings were taken using a digital monitor (ARSIMAI, BSX516, Munster, Germany). All procedures adhered to the standards outlined by the Canadian Society for Exercise Physiology (CSEP) [24].

After completing the consent and familiarization procedures, participants underwent orthostatic stress testing involving active standing, with continuous non-invasive beat-to-beat blood pressure monitoring via the Finometer™ Pro (Finapres Medical System BV, Amsterdam, The Netherlands). Upon conclusion of the assessment, participants were asked about any symptoms such as light-headedness or faintness to assess for orthostatic intolerance (OI). All assessments were conducted in a quiet, temperature-controlled environment (22.0 ± 1.0 °C), with humidity and barometric pressure kept at ambient conditions. To reduce the influence of circadian rhythms, testing was scheduled on weekdays between 8:30 and 11:30 a.m.

**Demographic, Anthropometric Measurements, and Frailty Index**

Demographic information, including sex assigned at birth, age, and date of birth, was collected, along with anthropometric and body composition data, to describe the characteristics of the study sample. Body mass (kg) was measured using the InBody 270 analyzer (©2015 InBody Co., Ltd., Cerritos, CA, USA), and height (m) was recorded using a SECA mobile stadiometer (SECA, Frankfurt, Germany). Frailty status was determined based on the Frailty Index (FI), which classified individuals as non-frail (≤0.08), pre-frail (>0.08 to 0.25), or frail (≥0.25), following established guidelines [23]. For instance, if a participant presents 10 deficits out of 30 possible indicators, the resulting FI would be 0.33, categorizing the individual as frail. A higher number of deficits corresponds to a greater degree of frailty.

**Assessment of Heart Rate Variability (HRV), Cardiac Parasympathetic Modulation (CPM), Cardiac Baroreceptor Gain (CBG)**

Short-term autonomic and cardiovascular responses were assessed through continuous beat-to-beat recordings of the electrocardiogram (ECG) and finger arterial pressure using the Finometer system (Finapres Medical System, Arnhem, The Netherlands) [25]. Data were collected with LabChart 8.0 software (ADInstruments, Colorado Springs, CO, USA) at a 1 kHz sampling rate and processed in MATLAB (R2024, The MathWorks Inc., Natick, MA, USA). Standard filtering, calibration, and signal-alignment procedures were applied to optimize R-R interval and systolic blood pressure (SBP) detection. Autonomic indices included HRV, CPM, and CBG. HRV was analyzed in the supine position using time-domain (mean R-R interval, standard deviation of RR intervals [SDNN], root mean square of successive differences between RR intervals [RMSSD]) and frequency-domain (low-frequency [LF], high-frequency [HF], and low-frequency to high-frequency power ratio [LF/HF ratio]) measures following international guidelines. CPM was derived from the HR 30:15 ratio of the minimum (longest RR-interval) around the 30th heartbeat and maximum HR (shortest RR-interval) around the 15th heartbeat after standing, representing cardiac vagal reflex function. CBG was calculated as the ratio of HR to SBP (ΔHR/ΔSBP, bpm/mmHg) at 30, 60, 180, and 420 s after active standing to assess dynamic baroreflex sensitivity. All measurements were obtained during sit-to-stand and lie-to-stand transitions at pre-test, 8 weeks, and 12 weeks of PST. For detailed instrumentation, calibration, and signal-processing procedures, see de Matos et al. [16].

**Physical Activity Levels, Hand Grip Strength, and Gait Speed**

Physical activity levels (PAL) were assessed using the International Physical Activity Questionnaire—Short Form (IPAQ-SF) [26], a validated self-report tool that estimates time spent on various intensities of activity over the previous seven days. The seven-item instrument captures frequency and duration of vigorous and moderate physical activity, walking, and sedentary time. Weekly physical activity was expressed in metabolic equivalent of task (MET) minutes by applying standard MET values. A Handful Hand Strength Dynamometer was used to measure the hand grip strength (HGS) following CSEP guidelines [24]. Participants completed two trials with their dominant hand, allowing 30 s of rest between attempts. The highest value was used for the analyses. To assess gait speed (GS), participants walked a 5-m flat, straight course at their usual pace, while the time taken was recorded. This procedure was repeated three times with 60-s breaks between trials. The mean of the three trials was used to calculate gait speed [24].

**Progressive Strength Training**

Participants engaged in the PST program that incorporated a series of exercises recommended by the Canadian Society for Exercise Physiology (CSEP) for older adults [24]. These exercises included leg press, leg extension, seated leg curl, chest press, shoulder press, seated rowing, calf raises, and cat-camel exercise. Program adherence and engagement were monitored using attendance logs and participant feedback, ensuring consistent participation throughout the intervention.

**Familiarization** **, 1 Repetition Maximum Test (1-RM), and Strength Training Intensity Control**

All participants underwent a structured familiarization process with the testing procedures, study protocols, and exercise training regimen. During the first three weeks, resistance loads were carefully introduced to ensure participants could safely engage in the PST program. Load determination was based on the one-repetition maximum (1-RM) test. Familiarization sessions were spaced approximately 48 to 72 h apart. The first session focused on introducing participants to the testing procedures and overall protocol, while the second session was dedicated to establishing individualized training loads for the PST intervention (see Table 1). This 12-week training followed exercise prescription principles as recommended by the CSEP [24] and the American College of Sports Medicine [27] guidelines. The intensity of the PST sessions was monitored using the OMNI-RES scale [28].

**Sample size**

The sample size was calculated using G*Power software version 3 (Heinrich Heine University, Dusseldorf, Germany) [29] based on an internal pilot study (*n* = 5). Power analysis was conducted using the F-test ANOVA repeated measures, within factors. For experiment 1 (sit-to-stand), an alpha of 0.05, with an actual power of 0.84 and an effect size of 0.53, based on a partial Eta-squared (η^2^p) value of 0.22. The values were observed in baroreceptor gain phase 1 (30 s) after standing during pre-test, 8 weeks, and 12 weeks in the pre-frail/frail group. The total sample size required for this experiment is 8 participants. For experiment 2 (lie-to-stand), an alpha of 0.05, with an actual power of 0.82 and an effect size of 0.62, based on a partial Eta-squared (η^2^p) value of 0.28. The values were observed in baroreceptor gain phase 1 (30 s) after standing during pre-test, 8 weeks, and 12 weeks in the pre-frail/frail group. The total sample size estimated for this experiment is 6 participants.

**Statistics**

The normality of the data distribution was evaluated using the Shapiro-Wilk test. Variables with a parametric distribution were expressed as mean ± standard deviation, with 95% confidence intervals (95% CI). Non-parametric data were presented as medians with interquartile ranges, along with the observed minimum and maximum values. For the sample characterization, continuous variables were summarized using descriptive statistics, including mean, standard deviation, and 95% CI, whereas categorical variables were described by absolute frequencies and percentages. For OI symptoms, given the nature of the study (single-group) and the binary outcome (yes/no symptoms), proportions were with exact binomial (Clopper-Pearson) 95% confidence intervals (CI), and changes over time were summarized using absolute risk differences with 95%CI calculated using the Newcombe method. To assess changes over three time points (pre-test, week 8, and week 12), a one-way repeated measures ANOVA was used for parametric data. For non-parametric variables, the Friedman One-Way repeated measures analysis of variance by ranks test was conducted. When statistically significant differences were identified, Bonferroni-adjusted *post-hoc* comparisons were applied. Cardiovascular medication use was included as a covariate in the main analyses to control for potential confounding effects on autonomic outcomes. To adjust for multiple hypotheses in CBG testing, the *p*-values were corrected using the Benjamini-Hochberg adjustment [30], with the false discovery rate set at 0.05. All statistical analyses were conducted using Jamovi (Version 2.3.28).

## 3. Results

Eight participants were enrolled in the PST program, consisting of 7 females (87.5%) and 1 male (12.5%), with a mean age of 70.2 ± 4.9 years and a mean height of 1.61 ± 5.9 m. Anthropometric variables (height, body mass, and BMI) and resting cardiovascular parameters (SBP, DBP, and HR) were similar across all three time points. The FI showed a significant reduction over time, with improvements in both 8 weeks and 12 weeks relative to the pre-test. However, no statistically significant change was observed between the 8- and 12-week assessments. In this pilot sample of older adults during the intervention period, the proportion of participants reporting OI symptoms decreased from 38% at baseline (3/8; 95% CI: 14–69%) to 13% at week 8 (1/8; 95% CI: 0.3–53%), with no participants reporting symptoms at week 12 (0/8; 95% CI: 0–37%). This represents an absolute reduction of 38 percentage points from baseline to week 12 (95%CI for risk difference ~6–65%). All individuals symptomatic at baseline (3/3) were symptom-free at study completion (Table 2).

**Frailty Index Changes Over the Progressive Strength Training**

**Figure 3:**
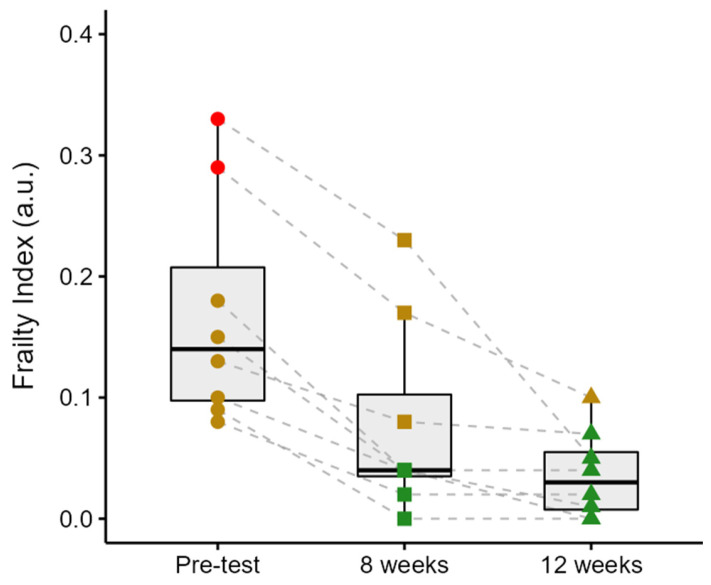
Progressive reduction in Frailty Index scores following 12 weeks of progressive strength training in pre-frail and frail older adults. Individual trajectories (dashed lines) and group distributions (box plots) show a significant decrease in FI from pre-test to 8 weeks and 12 weeks, indicating a transition from pre-frail/frail toward non-frail status. red dots = frail; yellow dots and squares and triangles = pre-frail, green squares and triangles = non-frail.

Figure 3 illustrates group and individual changes in the FI scores from the pre-test through the 12-week intervention period. The FI decreased by 56% from the pre-test to week 8 (*p* < 0.001), by 50% from week 8 to week 12 (although this did not reach statistical significance, *p* = 0.08), and by 78% from the pre-test to week 12 (*p* < 0.001). Participant 1 exhibited the highest initial FI (0.33), which decreased by 30% in week 8 (0.23) and further declined by 78% by week 12 (0.05). Participant 2 demonstrated a reduction from 0.29 to 0.17 (41%) over the first 8 weeks, followed by a decrease to 0.10 (41%) in week 12. Participant 3’s FI decreased from 0.18 to 0.04 (78%) in week 8 and remained stable thereafter. Participant 4 showed a progressive decline from 0.15 in the pre-test to 0.04 (73%) in week 8, and to 0.01 (75%) in week 12. Participant 5 improved from 0.13 to 0.08 (39%) by week 8 and to 0.07 (13%) by the end of the intervention. Participant 6 experienced a reduction from 0.10 to 0.04 (60%) after 8 weeks, ultimately reaching 0.00 in week 12. Participant 7 showed a complete reduction in FI from 0.09 to 0.00 within the first 8 weeks, with no further change observed. Lastly, Participant 8’s FI decreased from 0.08 to 0.02 (75%) in week 8 and remained unchanged thereafter. Two participants were classified as frail and six as pre-frail at baseline. By week 8, three participants were classified as pre-frail and five as non-frail, and by week 12, one participant was classified as pre-frail and seven as non-frail.

**Physical Activity Levels, Hand Grip Strength, And Gait Speed**

**Figure 4:**
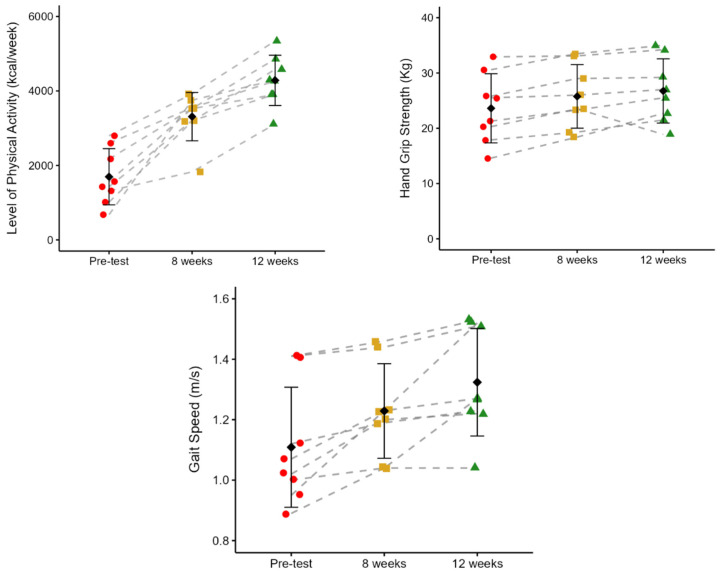
Changes in level of physical activity, handgrip strength, and gait speed following 12 weeks of progressive strength training (PST) in pre-frail and frail older adults. Data show individual trajectories (dashed lines), and group means ± SD across pre-test, 8 weeks, and 12 weeks. red dots = pre-test; yellow squares = week 8, green triangles = week 12.

Figure 4 shows the effects of PST on physical activity levels (PAL), hand grip strength (HGS), and gait speed (GS). Participants demonstrated a progressive, statistically significant increase in PAL over the 12 weeks of PST. The mean energy expenditure rose from 1695.34 ± 754.53 kcal/week at pre-test to 3310.39 ± 649.14 kcal/week at 8 weeks (95%; *p* = 0.002), and further to 4284.11 ± 675.48 kcal/week at 12 weeks (29%, *p* = 0.001). From pre-test to 12 weeks, there was a 153% increase in PAL (*p* < 0.001). HGS significantly increased from pre-test to both 8 and 12 weeks. There was an increase from 23.61 ± 6.25 kg at pre-test to 25.76 ± 5.75 kg at 8 weeks (*p* = 0.008), and to 26.75 ± 5.82 kg at 12 weeks (*p* = 0.046), representing a 9% and 13% improvement, respectively. However, no significant differences were observed between the 8 and 12 weeks of PST. From pre-test to 12 weeks, there was a 13% increase in HGS (*p* = 0.01). GS improved significantly after 12 weeks of PST. Pre-test GS was 1.11 ± 0.20 m/s, increasing to 1.23 ± 0.16 m/s at 8 weeks (11%, *p* = 0.018), and further to 1.32 ± 0.18 m/s at 12 weeks (7%, *p* = 0.017). No statistical differences were identified between 8 and 12 weeks (*p* = 0.1). From pre-test to 12 weeks, there was a 19% increase in GS (*p* = 0.01).

**Heart Rate Variability** **at Baseline in the Supine Position**

In the time and frequency domain analysis, no statistically significant differences were observed between pre-test, 8 weeks, and 12 weeks of PST (Table 3). When cardiovascular medication was included as a covariate in the analysis, the statistical results were not altered.

**Cardiac Parasympathetic Modulation (CPM)**

There were no statistically significant differences in CPM responses between pre-test, 8 weeks, and 12 weeks of PST (Table 4). Cardiovascular medication did not alter these results when it was included as a covariate in the analysis.

**Short-Term Baroreceptor Gain During Active Stand Orthostatic Stress Across Phases**

In the sit-to-stand and lie-to-stand transitions, no statistically significant differences were found in all phases between pre-test, 8 weeks, and 12 weeks of PST (Table 5). Cardiovascular medication use did not alter statistical results when it was included as a covariate in the analysis.

## 4. Discussion

To the best of our knowledge, this is the first study that analyzes the effects of 12 weeks of PST intervention on the phase-specific short-term autonomic compensatory responses in pre-frail and frail community-dwelling older adults under two distinct active-standing orthostatic stress conditions. Over the intervention period, improvements were observed in frailty index scores, physical activity levels, handgrip strength, and gait speed, and no participants reported orthostatic intolerance symptoms at week 12. In contrast, PST was not associated with improvements in autonomic function variables related to short-term compensatory responses.

**Frailty Dynamics in Response to 12 Weeks of Progressive Strength Training**

Throughout the 12 weeks of the PST intervention, participants demonstrated progressive improvements in frailty status, as evidenced by reductions in their FI scores. Overall, FI declined by 78% from baseline (pre-test), with marked improvements evident by week 8 and further reductions observed by week 12. Notably, the participant with the highest baseline frailty (FI = 0.33) showed the greatest improvement, transitioning from a frail to a non-frail status, with a reduction far exceeding the established minimal clinically important difference for the FI, which has been suggested to be approximately 0.03 points in previous literature [31,32]. This pattern aligns with prior evidence that individuals with higher baseline frailty (FI > 0.30) benefited the most from cardiac rehabilitation, with average FI reductions ranging from 0.08 to 0.11 [33] and among hospitalized frail older adults engaged in a supervised multi-component exercise program, decreasing from 0.26 ± 0.1 at admission to 0.20 ± 0.1 at discharge [4]. The observed reductions in frailty were accompanied by improvements in physical activity levels, handgrip strength, and gait speed, suggesting attenuation of physical frailty and enhanced functional capacity. These changes are plausibly mediated by neuromuscular and cardiovascular adaptations induced by PST, including improved muscle strength, mobility, and physiological reserve, which may also indirectly influence other frailty components such as fatigue and functional independence [34,35]. Moreover, the cumulative postural and muscular demands imposed by the training may have supported more efficient blood pressure regulation during orthostatic stress by enhancing baroreflex function and venous return [34,35]. Adaptations in peripheral circulation, including increased capillary density, may also have promoted more effective oxygen delivery and metabolic exchange during physical activity [34]. These systemic improvements may contribute to enhanced functional capacity and attenuation of physiological deficits associated with frailty, leading to enhanced functional independence and physiological reserve [36,37]. Collectively, these findings support the potential of PST to elicit meaningful improvements in frailty status, particularly among the most vulnerable older adults.

**Heart Rate Variability (HRV) Responses at Baseline After Progressive Strength Training**

No statistically significant changes were observed in HRV in either the time or frequency domains across the intervention. These findings are consistent with previous studies reporting no between-group differences in HRV following short-term strength training interventions in older adults [15,38]. Although Gambassi et al. [15] reported within-group improvements in HRV among trained participants, these changes were attributed to concurrent improvements in body composition and muscular strength, suggesting that autonomic adaptations may be mediated by reductions in body fat and increases in fat-free mass rather than strength training per se. Gerage et al. [38] found no significant HRV changes following 12 weeks of strength training in untrained older females compared to controls, proposing that limited training duration, volume and intensity may be insufficient to elicit autonomic modulation. In contrast, Lin et al. [14] observed improvements in HRV following 24 weeks of strength training in older adults, suggesting that longer intervention durations and higher training loads may be required to induce meaningful changes in vagal tone and orthostatic tolerance. Therefore, training load and duration seem to be critical for inducing autonomic adaptation. The absence of significant HRV changes in the current study may also reflect age- and frailty-related impairments in autonomic modulation. In pre-frail and frail older adults, autonomic function is commonly characterized by reduced parasympathetic activity and diminished reflex control of HR [39]. These alterations are frequently linked to structural and functional changes in the autonomic nervous system [12], including reduced baroreceptor sensitivity and altered central autonomic integration [12]. Such underlying physiological constraints may limit the responsiveness of cardiac autonomic modulation to short-term strength training interventions.

**Cardiac Parasympathetic Modulation (CPM) Responses During Active Standing Orthostatic Stress after Progressive Strength Training**

The CPM was similar across the pre-test, 8 weeks, and 12 weeks, with no statistically significant differences. The lack of statistical differences in CPM (HR 30:15 ratio) throughout the 12-week intervention suggests that the PST did not influence the vagal regulation of HR in this sample of pre-frail and frail community-dwelling older adults. Aging and frailty are commonly associated with a decline in parasympathetic activity [39], which reflects changes at both central and peripheral levels of autonomic control [12]. These include altered neurotransmission in brainstem nuclei involved in cardiovagal regulation, reduced acetylcholine availability at the sinoatrial node, and structural changes in afferent and efferent pathways that mediate parasympathetic output [39,40]. Such impairments may limit the ability of short-term interventions (≤ 12 weeks) to modify cardiovagal function and vagally mediated HR control, at least within the timeframe assessed in the current study (12 weeks).

**Short-term Baroreceptor Gain During Active Standing Orthostatic Stress after Progressive Strength Training**

CBG remained unchanged across pre-test, 8-week, and 12-week assessments, indicating no significant adaptation in baroreflex sensitivity over the course of the intervention. These findings are consistent with previous studies reporting no significant changes in CBG after short- to moderate-duration strength training interventions. For example, Fecchio et al. [18] observed no changes in CBG after 10 weeks of dynamic strength training in hypertensive males, suggesting that training duration and protocol characteristics may be insufficient to elicit baroreflex adaptations despite improvements in vascular function. Bellavere et al. [41] reported no significant changes in CBG after 16 weeks of strength training in older adults and proposed that limited sample size or reduced baroreflex responsiveness to resistance training may explain the absence of detectable effects. In pre-frail and frail older adults, attenuated baroreflex function is commonly reported and is associated with age-related alterations in arterial compliance, impaired afferent baroreceptor signaling, and changes in central autonomic integration [42,43]. These structural and functional alterations compromise heart rate regulation in response to blood pressure fluctuations, thereby limiting short-term autonomic control [43]. The lack of change observed in the present study may therefore reflect both the underlying autonomic impairments associated with frailty and the limited capacity of a 12-week PST intervention to modify the gain of the baroreflex arc in community-dwelling frail older adults. It is also possible that PST influenced the temporal characteristics of baroreflex responses rather than their magnitude. However, because CBG was assessed only at discrete time points following postural transitions (30, 60, 180, and 420 s), potential changes in the speed of baroreflex activation or recovery could not be captured. Future studies incorporating continuous or higher-resolution assessments may better elucidate whether resistance training affects the timing, rather than the amplitude, of short-term baroreflex compensation.

**Level of Physical Activity, Hand Grip Strength, and Gait Speed**

Throughout the 12-week PST intervention, participants exhibited gains in physical activity levels, hand grip strength, and gait speed, indicating improved functional performance and attenuation of physical frailty. Weekly physical activity increased by 153% from baseline to week 12, with substantial gains observed by week 8 and further increases thereafter. Handgrip strength improved by 9% at week 8 and by 13% at week 12, with no additional significant change between weeks 8 and 12. Similarly, gait speed increased by 11% at week 8 and reached an overall improvement of 19% by week 12, with no further significant gains during the final four weeks. The observed improvements in handgrip strength and gait speed are consistent with early-phase adaptations to resistance training, which are predominantly driven by neural mechanisms, including enhanced motor unit recruitment, increased firing rates, and reduced inhibitory signaling [34]. Over the course of the intervention, structural adaptations such as increased muscle fiber size and tendon stiffness may also have contributed to improved physical function [34,44]. In addition, the pronounced increase in physical activity levels likely reflects greater mechanical loading of the musculoskeletal system, reinforcing both neural and morphological adaptations that support improved motor performance in older adults [34,35].

**Orthostatic Intolerance Symptoms After Progressive Strength Training**

The proportion of participants reporting OI symptoms decreased from 38% at baseline (3/8) to 13% at week 8 (1/8), and to 0% at week 12, corresponding to an absolute risk reduction of 38 percentage points and a relative risk reduction of 100% from baseline to week 12. All participants who reported OI symptoms at baseline were symptom-free at week 12. The magnitude of risk reduction suggests potential clinical relevance, particularly given the association of OI with impaired functional independence and increased fall risk in frail older adults. Orthostatic intolerance in this population is commonly linked to impaired autonomic compensatory mechanisms during postural transitions, including reduced baroreflex sensitivity, attenuated vascular responsiveness, and delayed recovery of cardiac output and systemic vascular resistance [10,45], which are often exacerbated by frailty-related autonomic dysfunction [12].

**Limitation**

Several limitations of the present study should be acknowledged. First, cardiac baroreceptor gain (CBG) was assessed at discrete time points (30, 60, 180, and 420 s) during the active standing orthostatic stress protocol, which may not fully capture continuous or transient variations in baroreflex activity. While this approach provides insight into phase-specific short-term responses, it may overlook rapid adaptations or changes in the timing of baroreflex activation. Alternative methods, such as the sequence technique or transfer function analysis, which assess spontaneous systolic blood pressure–RR interval relationships and frequency-domain baroreflex behavior, respectively, may offer a more comprehensive characterization of baroreflex function [17].

Second, although the study met the minimum sample size estimated by the a priori power analysis, the inclusion of only eight participants limits generalizability, reduces statistical precision, and increases susceptibility to type II error. The small sample also constrained the ability to examine inter-individual variability and conduct subgroup analyses, such as stratification by frailty status or sex, which are known to influence autonomic and functional responses in older adults [46]. Relatedly, the pilot nature of the study limits definitive conclusions regarding the absence of change in autonomic indices, as subtle physiological adaptations may not have been detectable within this sample size or timeframe.

Third, the quasi-experimental time-series design without a randomized control group limits causal inference and increases the potential for bias relative to randomized controlled trials (RCTs) [46]. The absence of randomization may compromise internal validity and preclude direct attribution of observed changes to the intervention. Nevertheless, this design allowed for detailed longitudinal monitoring of individual responses, which is particularly relevant in heterogeneous and clinically complex populations such as pre-frail and frail community-dwelling older adults. Studies involving small but well-characterized cohorts can provide valuable physiological insights, especially in populations that are difficult to recruit and underrepresented in clinical research.

Future studies should build on these findings by employing adequately powered randomized controlled designs with longer intervention durations and continuous or high-resolution autonomic assessments to determine whether progressive strength training influences the magnitude and timing of baroreflex and autonomic compensatory responses, and to confirm the clinical relevance of the observed improvements in frailty and orthostatic intolerance outcomes.

## 5. Conclusions

In conclusion, frailty index scores decreased following 12 weeks of progressive strength training (PST), accompanied by improvements in physical activity levels, handgrip strength, and gait speed, suggesting attenuation of physical frailty in pre-frail and frail community-dwelling older adults. The descriptive and risk-reduction analyses indicated a progressive decline in the proportion of participants reporting symptoms over time. These findings should be interpreted cautiously and considered preliminary rather than confirmatory. In contrast, PST was not associated with improvements in variables related to short-term autonomic compensatory responses. Given the small sample size and absence of a control group, larger randomized controlled trials are warranted to confirm these findings and to determine whether longer or more intensive interventions are required to elicit autonomic adaptations.

## Figures and Tables

**Table 1:** Progressive strength training program progression.

Weeks	Frequency (days/week)	Loading (% RM)	Sets	Rest interval (sec)	Reps
1–3 (familiarization)	2	40–50% of 1RM	1	120	15
4–6	2	50–60% of 1RM	2	90	12
7–9	3	60–70% of 1RM	3	60	10
10–12	3	70–80% of 1RM	3	60	8

Reps = repetitions.

**Table 2:** Sample characterization.

Variables	Pre-Test		8 Weeks		12 Weeks		
Anthropometric	Mean ± SD	Min; Max	Mean ± SD	Min; Max	Mean ± SD	Min; Max	*p*
Body mass (kg)	75.3 ± 13.3	54.7; 91.8	75.4 ± 13.5	55.1; 92.6	77.4 ± 12.8	56.7; 92.1	0.5
Body mass index	28.8 ± 4.9	22.5, 36.3	28.9 ± 5.0	22.6, 36.6	29.0 ± 4.7	23.3, 36.4	0.5
Hemodynamic	Mean ± SD	Min; Max	Mean ± SD	Min; Max	Mean ± SD	Min; Max	
Resting SBP	132 ± 8.7	120; 141	128.0 ± 11.7	113; 149	128.5 ± 11.8	114; 148	0.1
Resting DBP	80 ± 11.0	60; 92	76 ± 11.7	61; 91	75 ± 7.4	64; 88	0.4
Resting HR	74 ± 8.2	64; 88	75 ± 8.1	62; 85	76 ± 4.0	68, 80	0.7
Frailty Status	Mean ± SD	Min; Max	Mean ± SD	Min; Max	Mean ± SD	Min; Max	*p*
Frailty Index	0.18 ± 0.1	0.08; 0.33	0.08 ± 0.04	0.00; 0.23	0.04 ± 0.04	0.00; 0.10	<0.001 *

* Statistically significant differences (*p* < 0.05).

**Table 3:** Heart Rate Variability (HRV) responses in time and frequency domains at rest across frailty groups at pre-test, 8 weeks, and 12 weeks.

Time Domain			
	Pre-test	8 weeks	12 weeks
RR	788.2 ± 300.7	814.2 ± 336.2	816.4 ± 365.0
	(557.0; 1019.3)	(533.2; 1095.2)	(478.8; 1153.9)
*p* (ES)	1.0 (0.1)b	0.9 (0.1)a	1.0 (0.1)c
SDNN	32.3 ± 15.5	27.6 ± 8.7	27.7 ± 5.1
	(20.4; 44.1)	(20.4; 34.9)	(23.0; 32.4)
*p* (ES)	0.4 (0.2)b	0.3 (0.2)a	1.0 (0.2)c
RMSSD	22.0 ± 7.3	19.7 ± 4.0	21.1 ± 6.3
	(16.3; 27.6)	(16.3; 23.0)	(15.3; 26.9)
*p* (ES)	1.0 (0.01)b	1.0 (0.01)a	1.0 (0.01)c
**Frequency Domain**			
LF	438.3 ± 650.7	207.9 ± 264.5	100.0 ± 59.1
	(−61.8; 938.5)	(−13.2; 429.0)	(45.4; 154.7)
*p* (ES)	0.9 (0.3) b	0.7 (0.3) a	1.0 (0.3) c
HF	915.0 ± 1818.0	426.4 ± 711.1	194.1 ± 114.2
	(−482.4; 2312.4)	(−168.2; 1020.9)	(88.5; 299.8)
*p* (ES)	1.0 (0.2) b	1.0 (0.2) a	1.0 (0.2) c
LF/HF	0.7 ± 0.4	0.7 ± 0.3	0.7 ± 0.4
	(0.4; 0.9)	(0.4; 1.0)	(0.3; 1.0)
*p* (ES)	1.0 (0.02) b	1.0 (0.02) a	1.0 (0.02) c

ES: Effect size, HRV: heart rate variability; RR: Average interval between consecutive heartbeats (RR-intervals); SDNN: Standard Deviation of RR intervals; RMSSD: Root Mean Square of Successive differences between RR intervals; LF: Low-Frequency power; HF: High-Frequency power; LF/HF: Low Frequency to High-Frequency power ratio: HR: heart rate. a. Statistical comparison between pre-test and 8 weeks; b. Statistical comparison between pre-test and 12 weeks; and c. Statistical comparison between week 8 and week 12. No statistically significant differences were observed for any variable.

**Table 4:** Cardiac parasympathetic modulation (HR Δ 30:15) during sit-to-stand and lie-to-stand transitions across frailty groups at pre-test, 8 weeks, and 12 weeks.

Cardiac Parasympathetic Modulation (CPM)			
**Sit-to-stand**			
	Pre-test	8 weeks	12 weeks
HR Δ 30:15	1.04|0.04	1.0|0.05	1.02|0.04
	(1.02; 1.18)	(0.93; 1.12)	(1.0; 1.17)
*p* (ES)	0.9 (0.01) b	0.8 (0.01) a	1.0 (0.01) c
**Lie-to-stand**			
	Pre-test	8 weeks	12 weeks
HR Δ 30:15	1.01 ± 0.06	1.04 ± 0.05	1.04 ± 0.04
	(0.91; 1.11)	(0.94; 1.12)	(1.0; 1.12)
*p* (ES)	0.6 (0.09) b	0.2 (0.09) a	1.0 (0.09) c

**|**: Interquartile range with (Minimum and Maximum). ES: effect size. a. Statistical comparison between pre-test and 8 weeks; b. Statistical comparison between pre-test and 12 weeks; and c. Statistical comparison between week 8 and week 12. No statistically significant differences were observed for any variable.

**Table 5:** Cardiac Baroreceptor Gain (CBG) responses during sit-to-stand and lie-to-stand transitions across frailty groups at pre-test, 8 weeks, and 12 weeks.

	Sit-to-stand		
CBG (bpm/mmHg)	Pre-test	8 weeks	12 weeks
Phase 1: 30 s	0.6|0.1	0.5|0.1	0.5|0.1
	(0.4; 0.8)	(0.4; 0.7)	(0.4; 0.6)
*p* (ES)	0.9 (0.6) b	0.7 (0.6) a	1.0 (0.6) c
Phase 2: 60 s	0.6|0.1	0.5|0.1	0.5|0.1
	(0.4; 0.8)	(0.4; 0.6)	(0.4; 0.7)
*p* (ES)	1.0 (0.6) b	0.2 (0.6) a	0.1 (0.6) c
Phase 3: 180 s	0.5|0.1	0.5|0.1	0.5|0.1
	(0.4; 0.8)	(0.4;0.7)	(0.3; 0.6)
*p* (ES)	1.0 (0.4) b	1.0 (0.4) a	1.0 (0.4) c
Phase 4: 420 s	0.6|0.1	0.5|0.1	0.5|0.1
	(0.4; 0.8)	(0.4; 0.7)	(0.4; 0.6)
*p* (ES)	1.0 (0.5) b	0.7 (0.5) a	1.0 (0.5) c
	Lie-to-stand		
	Pre-test	8 weeks	12 weeks
Phase 1: 30 s	0.6 ± 0.1	0.6 ± 0.1	0.6 ± 0.1
	(0.5; 0.7)	(0.5; 0.6)	(0.5; 0.7)
*p* (ES)	0.09 (0.1) b	0.1 (0.1) a	0.9 (0.1) c
Phase 2: 60 s	0.6 ± 0.1	0.6 ± 0.1	0.6 ± 0.1
	(0.5; 0.7)	(0.5; 0.7)	(0.5; 0.7)
*p* (ES)	1.0 (0.09) b	0.3 (0.09) a	0.1 (0.09) c
Phase 3: 180 s	0.6 ± 0.1 (0.5; 0.6)	0.6 ± 0.1 (0.5; 0.6)	0.6 ± 0.1 (0.5; 0.6)
*p* (ES)	1.0 (0.09) b	0.4 (0.09) a	0.2 (0.09) c
Phase 4: 420 s	0.6 ± 0.1	0.5 ± 0.1	0.6 ± 0.1
	(0.5; 0.6)	(0.5; 0.6)	(0.5; 0.7)
*p* (ES)	1.0 (0.07) b	0.5 (0.07) a	0.1 (0.07) c

|: Interquartile range with (Minimum and Maximum). CBG: Cardiac Baroreceptor gain; ES: Effect size. a. Statistical comparison between pre-test and 8 weeks; b. Statistical comparison between pre-test and 12 weeks; and c. Statistical comparison between week 8 and week 12. No statistically significant differences were observed for any variable.

## Data Availability

The raw data supporting the conclusions of this article will be made available by the authors on request.

## Abbreviations

ACSM	American College of Sports Medicine
ANS	Autonomic Nervous System
BP	Blood Pressure
CBG	Cardiac Baroreceptor Gain
CSEP	Canadian Society for Exercise Physiology
CPM	Cardiac Parasympathetic Modulation
DBP	Diastolic Blood Pressure
ECG	Electrocardiogram
ES	Effect Size
FI	Frailty Index
GS	Gait Speed
HG	Handgrip Strength
HF	High-Frequency Power
HR	Heart Rate
HRV	Heart Rate Variability
IPAQ-SF	International Physical Activity Questionnaire–Short Form
LFA	Low-Frequency Area (sometimes presented as LF)
LF	Low-Frequency Power
LPA	Level of Physical Activity
MCID	Minimal Clinically Important Difference
MET	Metabolic Equivalent of Task
OI	Orthostatic Intolerance
OH	Orthostatic Hypotension
OMNI-RES	OMNI Resistance Exercise Scale
PST	Progressive Strength Training
RMSSD	Root Mean Square of Successive Differences between RR Intervals
RR	Average Interval between Consecutive Heartbeats
SBP	Systolic Blood Pressure
SDNN	Standard Deviation of RR Intervals
TREND	Transparent Reporting of Evaluations with Non-Randomized Designs
